# Machine learning-based prediction of occupational exposure risks among oral healthcare workers

**DOI:** 10.3389/fpubh.2025.1713841

**Published:** 2026-01-05

**Authors:** Jinting Zhu, Lan Wang, Zhenjie Yu, Jingying Liu, Shuang Wu, Junxin Li, Dan Shan, Zhang Jian

**Affiliations:** 1 Department of Infection Management, Tianjin Stomatological Hospital, School of Medicine, Nankai University, Tianjin, China; 2 Tianjin Key Laboratory of Oral and Maxillofacial Function Reconstruction, Tianjin, China; 3 School of Nursing, Tianjin Medical University, Tianjin, China; 4 Diseases and Public Health, Jockey Club College of Veterinary Medicine and Life Sciences, City University of Hong Kong, Hong Kong, China; 5 Department of Prosthetics, Tianjin Stomatological Hospital, School of Medicine, Nankai University, Tianjin, China; 6 Painless Medical Center, Tianjin Stomatological Hospital, School of Medicine, Nankai University, Tianjin, China; 7 Department of Nursing, Tianjin Stomatological Hospital, School of Medicine, Nankai University, Tianjin, China; 8 Department of Oral Implantology, Tianjin Stomatological Hospital, School of Medicine, Nankai University, Tianjin, China

**Keywords:** occupational exposure, oral healthcare workers, machine learning, risk factors, predictive model

## Abstract

**Objective:**

This study aims to identify the key risk factors for occupational exposure among oral healthcare workers and develop a predictive model using machine learning algorithms to lay the foundation for early screening of high-risk populations and the formulation of preemptive intervention plans.

**Methods:**

A multicenter cross-sectional study was conducted among 367 oral healthcare workers in 27 hospitals in Tianjin, China, from January 2025 to June 2025. Data were collected via an online questionnaire, encompassing demographic information, Work Preference Inventory, Organizational Climates, resilience, and other relevant factors. Logistic regression, random forest, decision tree, and XGBoost algorithms were employed to construct predictive models. The models were evaluated based on the area under the receiver operating characteristic curve (AUC), accuracy, sensitivity, specificity, and F1 score.

**Results:**

The incidence rates of occupational exposure in the modeling and validation groups were 15.5% and 16.5%, respectively. Univariate analysis revealed significant differences between the exposed and non-exposed groups in terms of Work Preference Inventory, Organizational Climates, resilience, professional title, hospital level, age, and gender. Multivariate analysis using logistic regression indicated that Work Preference Inventory, resilience, Organizational Climates, professional title, hospital level, and gender were independent risk factors for occupational exposure. The random forest model exhibited the best predictive performance, with an AUC of 0.755, accuracy of 89.2%, sensitivity of 56.3%, specificity of 94.7%, and F1 score of 0.600.

**Conclusion:**

This study successfully identified the key risk factors for occupational exposure among oral healthcare workers and developed a predictive model using the random forest algorithm. These findings can guide the development of targeted interventions to mitigate the risks of occupational exposure. Future research should focus on validating the model with larger and more diverse datasets.

## 1 Introduction

Occupational exposure refers to the unintended contact of healthcare workers with pathogens, toxic or harmful substances, or sharp instruments during diagnostic, nursing, and related medical activities, which may lead to infection, poisoning, or other health damage. Depending on the source of exposure, occupational exposure can be classified into several types, including bloodborne exposure, chemical exposure, physical exposure, and biological exposure (1). According to data from the World Health Organization, healthcare workers are 2–19 times more likely to contract infectious diseases due to occupational exposure than other social groups. 40% of hepatitis B and C virus infections and 2.5% of human immunodeficiency virus (HIV) infections originate from occupational exposure. In recent years, with the increasing public awareness of oral health, the number of patients visiting dental clinics has been rising, imposing a heavier workload on oral health workers (2). As a high-risk department in medical services, the incidence of occupational exposure in oral medicine is also on the rise. During oral diagnosis and treatment, healthcare workers often use high-speed rotating instruments and sharp probes in confined spaces, and the patient's mouth contains a large number of contaminants, such as blood, saliva, and aerosols, which makes it highly possible for sharp instrument injuries, mucosal contact, and aerosol inhalation to occur (3). Moreover, dental treatment is time-consuming and requires delicate surgery. Under the conditions of long-term high-intensity work, healthcare workers are more likely to be distracted, further increasing the risk of exposure (4). As a high-risk department in medical services, the incidence of occupational exposure in oral medicine is also on the rise (5). Although previous studies have preliminarily explored the risk factors of occupational exposure for healthcare workers, traditional statistical methods have certain limitations in dealing with the interaction of multiple factors and nonlinear relationships, making it difficult to achieve accurate predictions. In addition, most previous studies have focused on a single type of exposure, lacking a comprehensive and systematic risk assessment tool for oral healthcare workers. With the development of artificial intelligence and machine learning technologies, it has become possible to construct occupational exposure risk prediction models based on multi-source data. Therefore, this study aims to identify the key risk factors of occupational exposure for oral healthcare workers and establish an occupational exposure risk prediction model suitable for them, based on existing literature and empirical data, by introducing algorithms such as logistic, random forest, decision tree, and XGBoost. By selecting the optimal model and improving prediction accuracy, this study provides theoretical support and practical guidance for occupational safety management and precise prevention and control.

## 2 Methods

### 2.1 Study population

This study is a multicenter cross-sectional study. A convenience sampling method was used to select healthcare workers from the oral departments of comprehensive hospitals and specialized oral hospitals in Tianjin who met the inclusion and exclusion criteria during the period from January 2025 to June 2025. Inclusion criteria: Healthcare workers in the oral department (including oral doctors, nurses, oral technicians, etc.) who hold a professional qualification certificate in oral medicine, nursing, or related fields and have been continuously on duty for 2 years before the survey date; those who voluntarily participated in this study. Exclusion criteria: Temporary transfer, visiting, or intern staff; those who were not on duty or unable to complete the questionnaire during the survey period. An online questionnaire survey was conducted using “Questionnaire Star.” All data included in this study were merged, and 70% of the study subjects (264 cases) were randomly selected as the modeling group, with the remaining part (103 cases) serving as the validation group. This study has been approved by the Ethics Committee (Approval No.:YPH2024-J-005), and all participants provided informed consent and voluntarily participated in this study.

### 2.2 Study instruments

#### 2.2.1 Demographic data

Based on literature review and expert consultation, a self-designed survey form for occupational exposure influencing factors among oral healthcare workers was developed. The general information survey form includes age, gender, occupational type, hospital level, hospital location, years of working in oral medicine, educational level, professional title, and other basic information.

#### 2.2.2 Work preference inventory (WPI)

This scale was translated into Chinese by Giovanni B based on the English version of WPI developed by Amabile in 1994 (6). The scale comprises two dimensions: intrinsic motivation and extrinsic motivation, with a total of 18 items. A 4-point rating scale is used, where 1 represents “strongly disagree” and 4 represents “strongly agree.” Higher scores indicate stronger work motivation among the subjects. The scale has good reliability and validity, with Cronbach's α coefficients for the two dimensions being 0.705 and 0.833, respectively (7).

#### 2.2.3 Organizational climates

The assessment of organizational climates was conducted using a four-dimensional Organizational Climates perception scale designed by Katz-Navon (8), which aims to measure healthcare workers' perceptions of safety-related policies, systems, and actual operations. The scale includes four dimensions: safety procedures, safety information flow, management safety practices, and safety priority, with a total of 21 items. A Likert 5-point scale is used for scoring, ranging from 1 (“strongly disagree”) to 5 (“strongly agree”). All four dimensions of Organizational Climates perception have good internal consistency, with Cronbach's α coefficients ranging from 0.85 to 0.89.

#### 2.2.4 Resilience

To evaluate resilience, the 10-item Connor-Davidson Resilience Scale (CD-RISC-10) was employed. This scale is derived from the original 25-item version, selecting 10 items that reflect the essential features of resilience. The CD-RISC-25, initially developed by Connor and Davidson, has been widely used to measure nurses' resilience levels and was later adapted for the Chinese context by Yu and Zhang. The CD-RISC-10 assesses the ability to cope with various situations, such as changes, personal challenges, and stress. Item responses range from 0 (“not true at all”) to 4 (“true nearly all the time”). The total score spans from 0 to 40, with higher scores indicating a stronger capacity for resilience. The validity and reliability of the CD-RISC-10 among the Chinese population have been well-established (9). The Cronbach's α coefficient for the CD-RISC-10 was 0.957.

### 2.3 Statistical analysis

**Figure 1:**
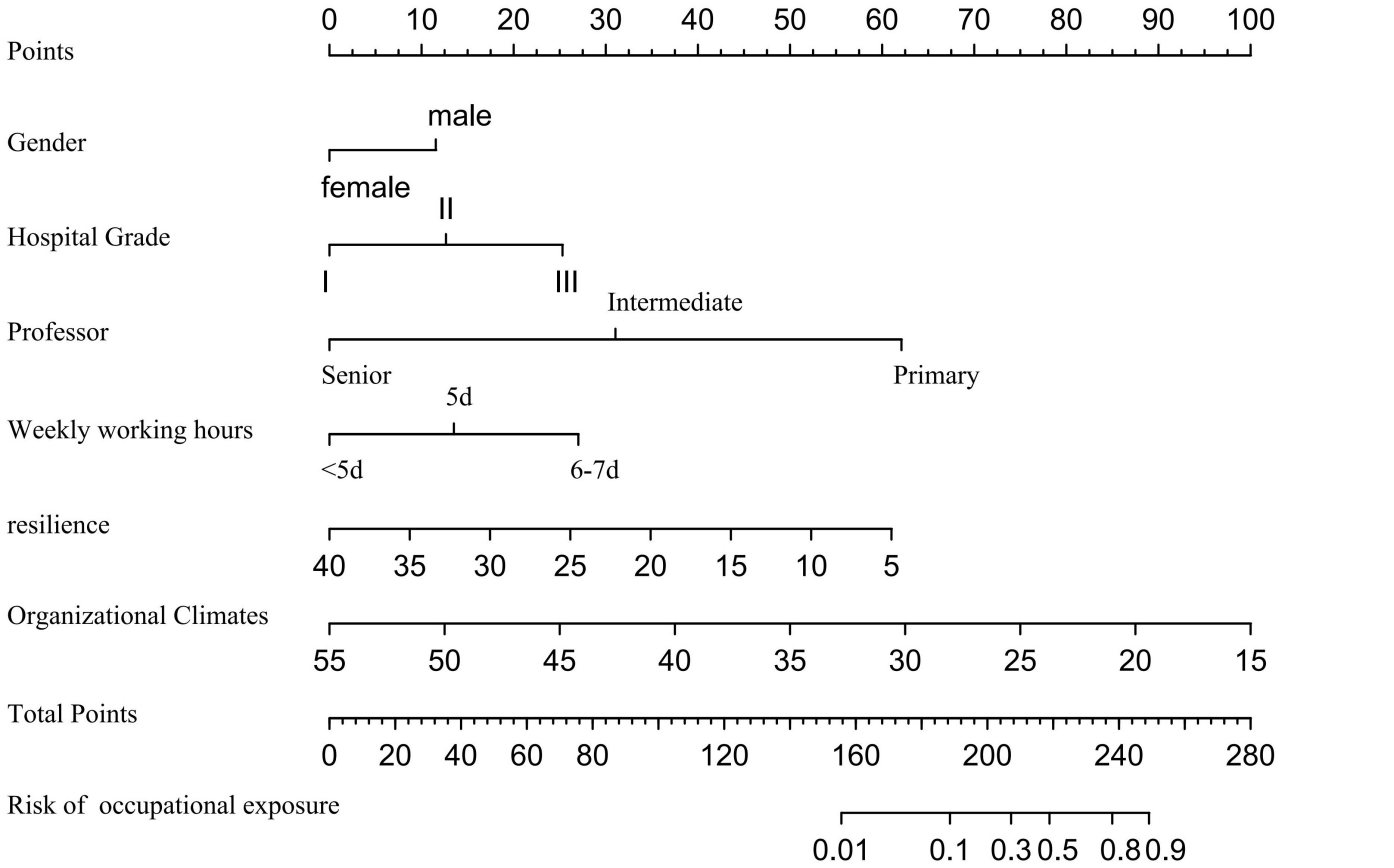
Nomogram for predicting occupational exposure risk among oral healthcare workers.

Statistical analysis was performed using R version 4.2.3. Normally distributed measurement data are presented as mean ± standard deviation ($\bar{\text{x}}$±s), and intergroup comparisons were made using t-tests. Non-normally distributed data are presented as median and interquartile range [M (QL, QU)], and intergroup comparisons were made using the Mann-Whitney U test. Count data are presented as frequency (n) and percentage (%), and intergroup comparisons were made using χ^2^ tests. Logistic regression was used to screen for influencing factors with occupational exposure as the outcome variable, and four predictive models were constructed based on this: Logistic Regression (LR), Random Forest (RF), Decision Tree (DT), and XGBoost. Nomogram for predicting occupational exposure risk among oral healthcare workers was shown in Figure 1. The predictive performance of the different models was compared using indicators such as the area under the receiver operating characteristic (ROC) curve (AUC), accuracy, sensitivity, specificity, and F1 score, with AUC values closer to 1 indicating better model predictive performance. A *P*-value of less than 0.05 was considered statistically significant.

## 3 Results

### 3.1 General information of study population

A total of 264 oral healthcare workers were included in the training set, among whom 101 (38.26%) were female healthcare workers, and 91 (34.4%) were aged under 30 years. In the modeling group, 41 healthcare workers experienced occupational exposure, with an incidence rate of 15.5%. The validation group included 103 healthcare workers, among whom 42 (40.7%) were female healthcare workers, and 41 (39.7%) were aged under 30 years. In the validation group, 17 healthcare workers experienced occupational exposure, with an incidence rate of 16.5%.

### 3.2 Univariate analysis of influencing factors

Univariate analysis was conducted on healthcare workers in the modeling group. The results showed that there were statistically significant differences (*P* < 0.05) between the two groups in terms of Work Preference Inventory, Organizational Climates, resilience, years of working in oral medicine, professional title, hospital level, age, and gender. However, no statistically significant differences (*P* > 0.05) were found between the two groups in educational level, occupational type, and hospital location. Specific data are shown in Table 1.

**Table 1:** Univariate analysis of influencing factors for occupational exposure among oral healthcare workers.

**Variable**	**Classification**	**Occupational exposure group(41)**	**Non occupational exposure group(233)**	**Z/χ*^2^***	***P***
Gender				4.716	**0.030**
	Male	16 (39.0)	95 (42.6)		
	Female	25 (61.0)	128 (57.4)		
Position				3.904	0.272
	Doctor	11 (26.8)	72 (32.3)		
	Nurse	28 (68.3)	127 (57.0)		
	Physician assistant	0 (0)	15 (6.7)		
	Technician	2 (4.9)	9 (4.0)		
Age				42.732	**<0.001**
	21–25	22 (53.7)	37 (16.6)		
	26–30	10 (24.4)	22 (9.9)		
	31–35	0 (0)	33 (14.8)		
	36–40	5 (12.2)	67 (30.0)		
	41–45	3 (7.3)	39 (17.5)		
	46–50	1 (2.4)	11 (4.9)		
	>50	0 (0)	14 (6.3)		
Hospital grade				10.910	**0.004**
	I	7 (17.1)	68 (30.5)		
	II	7 (17.1)	70 (31.4)		
	III	27 (65.8)	85 (38.1)		
Professor				45.589	**<0.001**
	Primary	37 (90.2)	75 (33.6)		
	Intermediate	2 (4.9)	104 (46.6)		
	Senior	2 (4.9)	44 (19.7)		
Place of residence				3.055	0.080
	City	38 (92.7)	182 (81.6)		
	Countryside	3 (7.3)	41 (18.4)		
Cultural level				0.413	0.854
	College diploma	21 (51.2)	123 (55.2)		
	Undergraduate degree	18 (43.9)	86 (38.6)		
	Graduate students	2 (4.9)	14 (6.3)		
Working years				8.968	**0.009**
	Less than 10 years	34 (82.9)	133 (59.6)		
	10-30 years	6 (14.6)	84 (37.7)		
	>30 years	1 (2.4)	6 (2.7)		
Organizational climates		46.0 (41.5, 54.5)	50.0 (43.0, 70.0)	11.051	**0.004**
Resilience		22 (19.5, 26.5)	28 (21, 33)	0.897	0.639
Work preference inventory		21.0 (21.0, 24.5)	23.0 (21.0, 27.0)	32.169	**<0.001**

Bold indicates *p* < 0.05.

### 3.3 Selection of input variables

The eight variables that were statistically significant in the univariate analysis were included as independent variables in the four models, with occupational exposure as the dependent variable for the analysis of influencing factors. The coding methods for the independent variables included in the model are as follows: Age: 21–25 = 1, 26–30 = 2, 31–35 = 3, 36–40 = 4, 41–45 = 5, 46–50 = 6, >50 = 7; Working years: Less than 10 years = 1, 10–30 years = 2, >30 years = 3; Professional title: Junior title = 0, Intermediate title = 1, Senior title = 2; Gender: Female = 0, Male = 1; Hospital level: Level 1 hospital = 1, Level 2 hospital = 2, Level 3 hospital = 3. Work Preference Inventory, Organizational Climates, and resilience are continuous variables and were entered into the model using their original values. The results of the multivariate analysis are shown in Table 2.

**Table 2:** Multivariate analysis of influencing factors for occupational exposure among oral healthcare workers.

**Variable**	**β**	**SE**	**OR (95%CI)**	***P***
Work preference inventory	−0.122	0.060	0.885 (0.787, 0.995)	**0.040**
Resilience	−0.135	0.035	0.874 (0.816, 0.936)	**<0.001**
Organizational climates	0.005	0.021	1.005 (0.965, 1.047)	0.797
Working years	−1.141	0.541	0.320 (0.111, 0.922)	**0.035**
Professor	−2.213	0.687	0.109 (0.028, 0.420)	**0.001**
Hospital grade	−1.363	0.354	3.908 (1.951, 7.827)	**<0.001**
Age	−0.454	0.266	0.635 (0.377, 1.069)	0.635
Gender	−1.497	0.518	4.468 (1.617, 12.344)	**0.004**
Constant	−0.822	0.765	0.439 (0.098, 1.968)	0.282

Bold indicates *p* < 0.05.

### 3.4 Construction of predictive models

**Figure 2:**
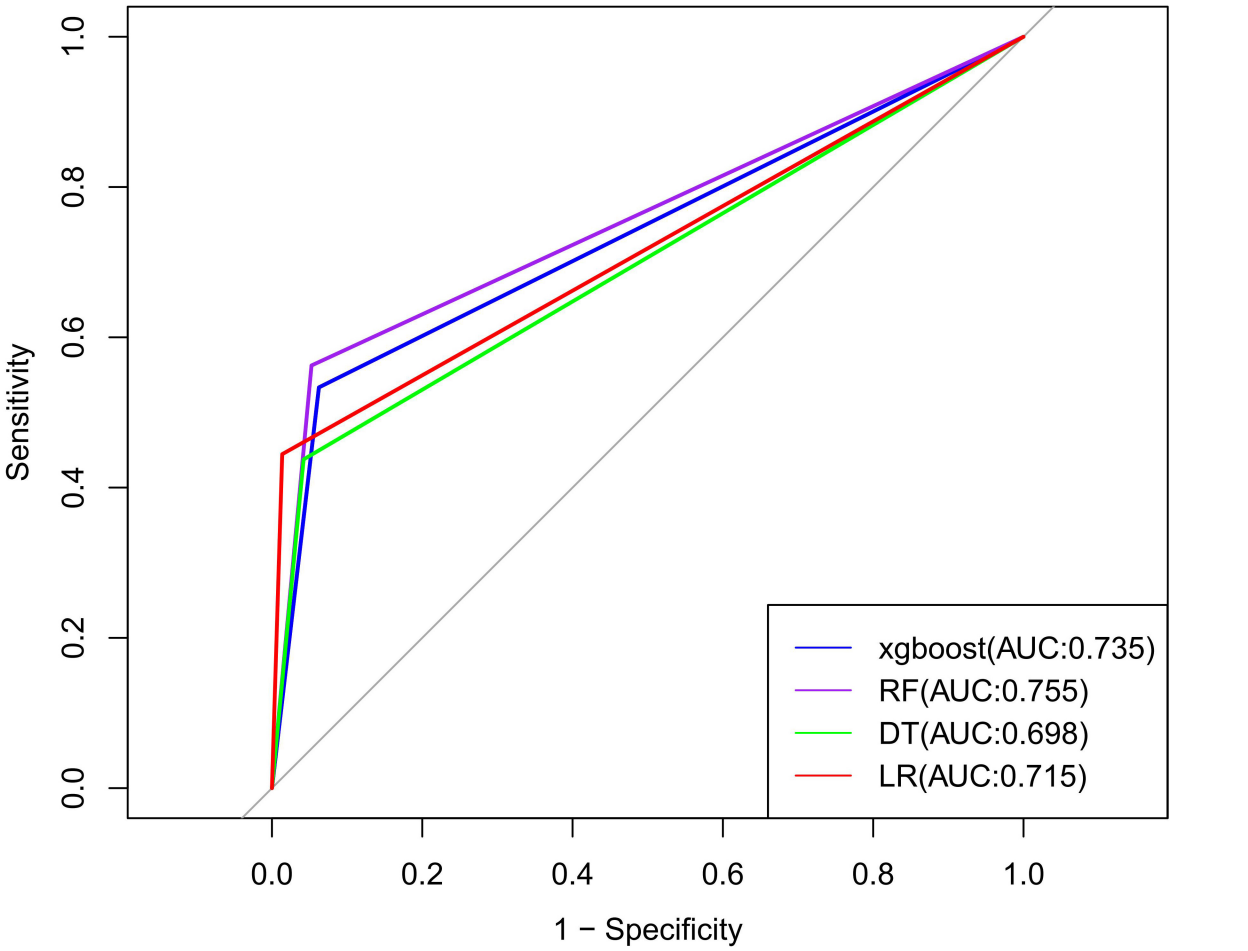
Comparison of ROC curves for different machine learning models.

**Figure 3:**
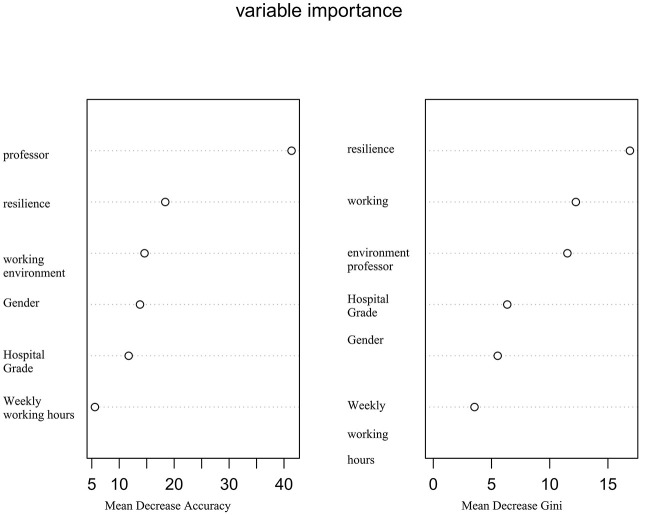
Variable importance in random forest model.

The models were constructed using the R language. With six risk factors in the training set as independent variables and occupational exposure as the dependent variable, the following models were trained: logistic regression, random forest algorithm, decision tree algorithm, and XGBoost algorithm. Grid search and five-fold cross-validation were used to determine the optimal parameters for each model. After the models were established, the validation set data were used to validate each model, and the AUC, sensitivity, specificity, and Youden's index of each model were calculated. The results showed that the random forest model had the highest AUC, while the DT algorithm model had the lowest AUC; the random forest model had the highest sensitivity and the highest specificity, while the decision tree algorithm model had the lowest specificity, which shown in Figure 2. Specific results are shown in Table 3. By using the Feature Importances method of the Random Forest, the importance weights of each feature variable are listed in order, as shown in Figure 3.

**Table 3:** Comparison of predictive performance of different models.

**Model**	**ROC**	**Accuracy**	**Sensitivity**	**Specificity**	**Pos pred value**	**Neg pred value**	**F1**
LR	0.715 (0.633–0.799)	0.910	0.444	0.986	0.842	0.916	0.582
DT	0.698 (0.571–0.825)	0.883	0.438	0.958	0.636	0.910	0.519
XgBoost	0.735 (0.603–0.869)	0.883	0.533	0.938	0.571	0.928	0.552
RF	0.755 (0.627–0.883)	0.892	0.563	0.947	0.643	0.928	0.600

## 4 Discussion

### 4.1 High risk of occupational exposure among oral healthcare workers

The results of this study indicate that the risk of occupational exposure among oral healthcare workers is relatively high. This finding is similar to the results of the survey on occupational exposure among healthcare workers by Shi Yu (10), but higher than the results of the survey on occupational exposure among operating room healthcare workers by Sun Xiaoyan (11). The differences in the survey populations may account for these variations. First, during oral diagnosis and treatment, healthcare workers frequently use high-speed rotating instruments for dental restoration, tooth extraction, and other procedures, generating a large amount of aerosols and splashes containing patients' blood, saliva, and microorganisms (12). These aerosols can remain suspended in the air for a long time and may even penetrate protective equipment such as masks and goggles, thereby increasing the risk of healthcare workers inhaling pathogens. Additionally, the confined space of oral operations makes it difficult for healthcare workers to maintain a sufficient safety distance from patients, further increasing the risk of occupational exposure (13). Second, although healthcare workers are required to wear protective gear such as masks, goggles, and gloves during oral procedures, the use of goggles can impair the operator's vision, and gloves can reduce finger dexterity. As a result, some healthcare workers may choose not to wear or improperly wear protective equipment during operations. Moreover, prolonged use of protective gear can lead to fatigue, reducing the effectiveness of protective measures and significantly increasing the risk of occupational exposure. To reduce the risk of physical and mental harm to oral healthcare workers, the government should invest time, resources, and effort in providing a favorable environment for these professionals (14). In addition, oral healthcare workers themselves should continue to enhance their communication skills with patients to prevent occupational exposure and protect their physical and mental health. Hospitals should also actively monitor the psychological changes in healthcare workers following exposure incidents and promptly provide psychological support and intervention to safeguard their well-being.

### 4.2 Higher risk of occupational exposure among female oral healthcare workers

The results of the logistic regression model show that female oral healthcare workers have a higher risk of occupational exposure (OR = 4.468, *P* = 0.004), which is consistent with the findings of Collins C (15). Compared with male healthcare workers, female healthcare workers generally have shorter arm lengths. When performing oral procedures, they need to be closer to the patient's mouth to ensure the accuracy of the operation. This close proximity increases the risk of contact with the patient's blood, saliva, and splashes, thereby raising the risk of occupational exposure (16). Second, female healthcare workers tend to pay more attention to patients' emotional needs during work, sometimes neglecting their own occupational protection. When facing patients' emotional fluctuations or sudden situations, female healthcare workers may be more willing to actively contact patients to provide emotional support, which may increase the risk of occupational exposure. Moreover, during pregnancy or lactation, female healthcare workers may experience changes in body shape, further affecting the fit of protective equipment. Additionally, the increased frequency of using the restroom during pregnancy and lactation leads to more frequent donning and doffing of protective gear, reducing the continuity and effectiveness of protective measures and increasing the risk of occupational exposure.

### 4.3 Higher risk of occupational exposure among healthcare workers with higher hospital level and lower professional title

The results of the logistic regression model indicate that healthcare workers in higher-level hospitals and those with lower professional titles have a higher risk of occupational exposure (OR = 3.908, *P* < 0.001; OR = 0.109, *P* = 0.001). This finding is inconsistent with that of Yuanshuo Ma (17), but consistent with that of Afia Zafar (18). Higher-level hospitals are equipped with more advanced diagnostic and therapeutic devices and have more complex procedures, which increase the risk of contact with splashes or aerosols during surgery. In addition, the patient flow in higher-level hospitals is greater, and the workload of healthcare workers is higher. Long working hours can lead to fatigue and inattention, further increasing the risk of occupational exposure (19). Healthcare workers with lower professional titles (such as junior nurses and resident physicians) are usually in the early stages of their careers and lack rich clinical experience and the ability to handle complex situations (20). When facing high-risk diagnostic and therapeutic operations, they may not be able to quickly identify potential occupational exposure risks and take effective protective measures. Moreover, healthcare workers with lower professional titles are usually responsible for more basic nursing tasks, such as cleaning, dressing changes, and blood drawing, which have a higher likelihood of direct contact with patients, further increasing the possibility of occupational exposure. In addition, Guo Xiaoying (21) believe that although the risk index and difficulty coefficient of the tasks undertaken by healthcare workers increase with the professional title, the occupational exposure risk of healthcare workers with intermediate professional titles is higher than that of senior titles. This is because intermediate title personnel have a broader scope of work, more arduous tasks, and more frequent contact with patients in practice, leading to a significantly higher risk of occupational exposure. Therefore, it is of great significance for the country to fundamentally solve the problem of the shortage of healthcare workers in Chinese hospitals, strengthen occupational protection training for healthcare workers with lower professional titles, optimize the design of protective equipment, improve the effectiveness of protective measures, and improve the working environment to reduce the risk of occupational exposure.

### 4.4 Higher risk of occupational exposure among oral healthcare workers with low resilience

The results of this study show that oral healthcare workers with low resilience have a higher risk of occupational exposure. Resilience (22) refers to an individual's ability to effectively cope with and recover from stress, adversity, or trauma. Studies have shown that healthcare workers with stronger resilience have a lower risk of occupational exposure (22). The reason is that healthcare workers with strong resilience can more effectively identify potential occupational exposure risks when facing complex working environments and high-risk medical operations. When encountering sudden situations, they can quickly adjust their mindset and avoid neglecting protective details due to emotional fluctuations, thereby reducing the risk of occupational exposure (23). Moreover, healthcare workers with strong resilience have a stronger sense of self-efficacy and self-protection. They are more inclined to actively acquire occupational protection knowledge, actively participate in relevant training, understand the transmission routes and protection points of different pathogens, and strictly follow operating procedures in actual work, thereby reducing the risk of occupational exposure (24). In addition, oral healthcare workers with strong resilience can better regulate their own stress and avoid fatigue and inattention caused by long-term high-pressure states, thereby reducing the risk of occupational exposure due to negligence or fatigue.

### 4.5 Lower risk of occupational exposure among oral healthcare workers with strong work motivation and long working experience in oral medicine

The results of this study show that oral healthcare workers with strong work motivation and long working experience in oral medicine have a lower risk of occupational exposure. Work motivation refers to the level of proactivity and engagement that an individual demonstrates in their work (25). Healthcare workers with high work motivation can more effectively utilize available resources when facing occupational exposure risks. They will actively seek support and help from colleagues, promptly report potential occupational exposure events, and handle them according to the hospital's procedures (26). Moreover, healthcare workers with high work motivation usually have a stronger sense of professional identity and belonging. They are more willing to invest more energy in their work, actively improve their professional skills and occupational literacy, and view issues from the perspective of career development. When facing complex patient conditions or high-risk medical operations, healthcare workers with high work motivation can quickly adjust their work strategies to avoid psychological stress and occupational exposure risks caused by environmental changes (27, 28). In addition, healthcare workers with long working experience have accumulated more clinical experience, enabling them to more accurately identify potential occupational exposure risks and take effective protective measures when facing high-risk medical operations.

### 4.6 Analysis of the value of the occupational exposure risk prediction model for oral healthcare workers

In this study, we constructed a risk prediction model based on six selected variables using a variety of machine learning algorithms. Comparing the performance of different models, we found that the Random Forest model demonstrated the best efficacy. This model has high accuracy, sensitivity, and specificity. Moreover, the prediction model is simple and easy to use, making it highly practical. It allows clinical managers to quickly identify oral healthcare workers at higher risk of occupational exposure, enabling early intervention to mitigate the adverse effects of occupational exposure. The Random Forest model's superior performance can be attributed to several factors. Firstly, it effectively handles high-dimensional data and is robust against overfitting, a common issue in models with complex datasets. Secondly, it naturally addresses data imbalances through bootstrapping and subsampling, which is particularly beneficial in medical datasets where certain outcomes may be rare. Lastly, the model's ability to capture non-linear relationships and interactions between variables provides a more nuanced understanding of the data, leading to more accurate predictions. We employed a rigorous cross-validation strategy to validate our model, ensuring that our findings are generalizable and not a result of overfitting to a specific subset of data. This methodological approach significantly strengthens the credibility of our study and the reliability of the Random Forest model as a predictive tool in this context. By explicitly justifying the Random Forest model's performance relative to other models and discussing potential challenges such as overfitting and data imbalance, we provide a clear and comprehensive rationale for our choice of model. This study not only enhances the methodological rigor but also contributes to the practical application of machine learning in occupational health risk prediction, offering a robust tool for early identification and intervention.

## 5 Conclusion

This study constructed a risk prediction model for occupational exposure among oral healthcare workers using multiple machine learning algorithms. After evaluation and comparison, the Random Forest model was identified as the optimal prediction model. The risk factors for occupational exposure among oral healthcare workers include Work Preference Inventory, resilience, years of working in oral medicine, professional title, hospital level, and gender. Clinical managers should develop and implement intervention plans targeting these risk factors to reduce the harm of occupational exposure to oral healthcare workers. This study acknowledges its limitations due to its cross-sectional design and being conducted exclusively in oral healthcare institutions in Tianjin, which may not fully account for unmeasured hospital-specific policies or cultural factors that could influence outcomes, potentially introducing biases. The Random-Forest-based prediction model for occupational exposure among oral healthcare workers still requires large-scale surveys for further refinement and validation.

## Data Availability

The raw data supporting the conclusions of this article will be made available by the authors, without undue reservation.
