# Correction: Machine learning-based prediction of occupational exposure risks among oral healthcare workers

**DOI:** 10.3389/fpubh.2026.1781802

**Published:** 2026-02-10

**Authors:** Jinting Zhu, Lan Wang, Zhenjie Yu, Jingying Liu, Shuang Wu, Junxin Li, Dan Shan, Zhang Jian

**Affiliations:** 1 Department of Infection Management, Tianjin Stomatological Hospital, School of Medicine, Nankai University, Tianjin, China; 2 Tianjin Key Laboratory of Oral and Maxillofacial Function Reconstruction, Tianjin, China; 3 School of Nursing, Tianjin Medical University, Tianjin, China; 4 Diseases and Public Health, Jockey Club College of Veterinary Medicine and Life Sciences, City University of Hong Kong, Hong Kong, China; 5 Department of Prosthetics, Tianjin Stomatological Hospital, School of Medicine, Nankai University, Tianjin, China; 6 Painless Medical Center, Tianjin Stomatological Hospital, School of Medicine, Nankai University, Tianjin, China; 7 Department of Nursing, Tianjin Stomatological Hospital, School of Medicine, Nankai University, Tianjin, China; 8 Department of Oral Implantology, Tianjin Stomatological Hospital, School of Medicine, Nankai University, Tianjin, China

**Keywords:** occupational exposure, oral healthcare workers, machine learning, risk factors, predictive model

Authors Jinting Zhu, Zhenjie Yu, Jingying Liu, Shuang Wu, Junxin Li, and Dan Shan were erroneously published as Zhu Jinting, Yu Zhenjie, Liu Jingying, Wu Shuang, Li Junxin, and Shan Dan.

The order of authors in the author list of the published paper was erroneously given as:

Zhang Jian^*^, Zhu Jinting^†^, Lan Wang^†^, Yu Zhenjie, Liu Jingying, Wu Shuang, Li Junxin and Shan Dan

The correct author list reads:

Jinting Zhu^†^, Lan Wang^†^, Zhenjie Yu, Jingying Liu, Shuang Wu, Junxin Li, Dan Shan and Zhang Jian^*^

^†^These authors have contributed equally to this work

^*^Corresponding author

The original version of this article has been updated.

